# Editorial

**Published:** 1847-10

**Authors:** 


### THE MEDICAL EXAMINER.

### PHILADELPHIA, OCTOBER, 1847.

### EPIDEMIC CEREBRO-SPINAL MENINGITIS IN MISSOURI.

In our number for August last, (p. 506,) we inserted in the 'Record' department, a communication, addressed to the *New Orleans Medical and Surgical Journal*, on the subject of a highly fatal disease, which prevailed in the early part of the year in Mississippi and Tennessee, and was manifestly an affection of the same character as one which visited with fearful malignity different towns of France, attacking principally the common soldiers of the garrisons. It was seen also in Ireland in 1846; and everywhere has presented the same great general characters. The term *cerebro-spinal* seems to be applied to it with propriety, and it evidently attacks, in a marked manner, the gray matter in the centre of the cord—the nervous system of reflex actions. It would appear from the following extract of a letter, addressed to Professor Dunglison by Dr. W. C. Philips, dated Rocheport, Boon county, Missouri, September 13th, 1847, that the scourge has been devastating localities widely apart from each other, and it is not improbable that it may extend elsewhere.

"I take the liberty," says Dr. Philips, in his intelligent epistle, "of advising you of the prevalence of a disease, that has excited considerable interest in this country, from its nondescript and fatal character.

"*Symptomatology*. There are no invariable symptoms attending it. Generally, the patient has been in ordinary health, pursuing his accustomed occupation, and the first indication he has of the approach of the disease is, perhaps, a pain in the hand, foot, arms, legs, eye, brain, lungs, stomach, or bowels. Any one, or a number of these locations in connection, may be primarily affected—sometimes there is a sensation of cords drawing at the back of the neck, producing stiffness. In one case, there was contraction of the occipito-frontalis, and the muscles of the face. In many cases, there is so much soreness of the surface, as to render the smallest amount of clothing intolerable. It is usually ushered in with a chill of variable intensity, succeeded by similar reaction, and this reaction is followed in from 3 to 12 hours by a second chill, and if disorganization has not previously taken place, (which is often the case) it appears to accompany, or immediately succeed it. In many cases, there is excruciating pain in the arms, legs, and other parts of the body, and this pain, when located in the limbs, is often accompanied by swelling of the joints and more or less loss of the use of the limbs, with stiffness and inability to move them. These pains are sometimes permanent, but at others, shifting from one point to another. The neck is often drawn backwards or forwards at an angle of almost 45° from its natural position, and so rigidly fixed, that it would break it short off to force it to resume its physiological position, and is swollen very much larger than its ordinary size. The external surface is sometimes beautifully spotted; and in a few cases there was an exanthematous eruption. The nervous system is deeply involved. There may be profound coma,—wild and furious delirium,—subsultus tendinum, &c., apoplexy and paralysis. In one case, there was blindness of one eye, accompanied with permanent pain in the head; also swollen joints, (terminated fatally.) Lungs may or may not be implicated. Stomach the seat often of nausea and vomiting. Bowels, nothing characteristic. In a few cases, there was gastro-enteritis. Trismus occurred in one case. In other cases, there was an inability to swallow any thing, from the swollen and painful condition of the larynx and pharynx.

"*Etilogy*. Most common form 10 to 15 years of age; but occurs at all periods of life. It resembles in many respects the prevailing disease of our country, (at the time of its occurrence last spring,) which was a modified form of pneumonia. There was, in both, the same soreness of the surface. The chill ushering in; the pneumonic symptoms and the swelling of the joints were similar; and the worst forms of the prevailing disease, and this malignant scourge, were similar in their duration, termination, &c. From these facts I infer, that they are produced by the same general cause, and that there exist local causes where it prevailed giving its malignant type. The disease is confined to a section of country in the Missouri River bottom, which was the seat of a great overflow in June, 1844, at which time an extensive layer of sand was deposited upon the soil, entombing large crops of vegetable matter. After the overflow, this bottom has been unusually healthy until last spring, whilst the rest of our country has suffered far more from disease than usual. Would it be philosophical or scientific to say this overflow produced these local causes?

"*Prognosis* is unfavourable: five-sixths die. In many cases, it is death *ab initio*. When fatal, it is generally so in from six hours to two days."

---

### DR. RUSCHENBERGER, U. S. N.

This able medical officer, whose services in the Navy have been long highly appreciated, not only when afloat, but in the naval hospitals of the country, and who has been not less distinguished for his incessant efforts to elevate the character of the corps, and to obtain for its officers—in which he succeeded—a fixed rank in the Navy, has left the Naval Hospital, New York, to reside in the place of his nativity—Philadelphia. Of that hospital he has had the professional care for four years; and has been succeeded by Surgeon Waters Smith.

The following—we believe, unusual—testimonial is honourable to the giver and the receiver, and we have no doubt was as true as it is honourable; and, although at the risk of offending the modesty of the party most concerned, we publish it as an incentive to others to merit the same tribute.

NAVY DEPARTMENT,
*Bureau of Medicine and Surgery, Sept. 2d,* 1847.

*Sir*,—I have to inform you, that the Board of officers, ordered on the 16th inst. to examine into the state and condition of the Naval Hospital at Brooklyn, then under your charge, have submitted their report to this office. The character of this report is so highly complimentary to yourself, I deem it proper to furnish you with an extract, as follows:—"The order, method, cleanliness and good discipline everywhere apparent, from the sick wards to the most inferior offices, were highly satisfactory. The exact system of accounts deserves to be particularly noticed as an act of justice to the surgeon, who has devoted great pains and much labour in the details.

"Unusual attention was observable in the Laboratory Department, which has been turned to a good account, in an economical view, in the preparation of many articles of materia medica, as well as securing uniformity in the strength of various preparations."

I have the pleasure to add, that I am authorized to signify to you the high satisfaction entertained by the Department for the very able manner in which all your duties have been performed while Surgeon in charge. Respectfully,

THOMAS HARRIS,
per J. L. FOX, Assistant to Chief.

DR. W. S. W. RUSCHENBERGER, Surgeon, U. S. Navy, Brooklyn, N. Y.

### DOCTOR ANDREW COMBE.

[We are indebted to a friend for the following notice of Dr. Combe, which will be read with interest, especially by those who are conversant with the admirable productions of his pen.]

Of the death of Dr. Combe, our readers have been apprised through the public papers. On us devolves the duty of indicating the special merits of our deceased brother, and the loss to the profession by his death. Here, if any intelligent reader were at our side, he might interrupt us by remarking on the signal services rendered to mankind by the writings of Dr. Combe, and allege that still, in a greater degree, must his loss be felt by the members, generally, of every community, than even by his professional brethren. Literally, and in its full meaning, may it be said, that man, woman and child, are under the strongest obligations to this popular writer, for his singularly lucid and practical teaching of hygiene, in connexion with physiology, as evinced in his different works, of which we shall soon speak under their respective titles. In another point of view, the labours of Dr. Combe furnish a lesson of encouragement to those who, owing to failing health, might be deterred from prosecuting their literary and professional labours, and of reproof to others who plead every slight indisposition and mental depression as excuses for not discharging their duties in these respects. He became a victim to the inroads of pulmonary consumption soon after the age of manhood; but yet he continued to be the zealous student, and at intervals the industrious and successful writer, during the whole of the period between the date of his first attack of disease, in 1820, and that of his death, on the 9th of August, 1847, a little before he had attained his fiftieth year.

Doctor Combe was born in Edinburgh on the 27th of October, 1797, being the fifteenth child, and seventh son, of parents whose entire progeny numbered seventeen. Having gone through the usual course of instruction at the High School, he was bound apprentice, in conformity with the usage in Great Britain, to the late Henry Johnston, Esq., surgeon in Edinburgh, and in 1817 he had acquired an amount of knowledge which entitled him to be licensed as a surgeon. With the view of farther qualifying himself for medical parctice, he next repaired to Paris, where two years were laboriously spent in attending on the hospitals and listening to the instruction so prodigally given out in the lectures of the many professional celebrities of that capital. In 1823 he began to practice in Edinburgh, and about two years later took there his medical degree. Success attended his professional labours, marked as they were by sagacity, kindliness and conscientiousness; and he was in the course of a few years in the enjoyment of a flourishing practice. But a return of symptoms of pulmonary disease obliged him, in 1831, to proceed to Italy, where he had been once before from a similar cause. He was, however, able to pass the winter of 1832–3 in Scotland, and in the latter year to resume his practice. "In 1836 he was honoured with the appointment of Physician in Ordinary to the King and Queen of the Belgians, and for several months attended the royal family in Brussels; but the climate proving unfavourable to him, an alarming return of his pulmonary symptoms abruptly sent him back to recruit his health in his native land. Subsequently he continued to act as consulting physician to their Majesties, and occasionally paid them a visit. About six or seven years ago, he was appointed one of the Physicians Extraordinary to the Queen in Scotland, and afterwards one of her Majesty's physicians in Ordinary in this part of the united kingdom. He was, also, a Fellow of the Royal College of Physicians of Edinburgh, and a corresponding member of the Imperial and Royal Society of Physicians of Vienna."

The works by which the name of Dr. Combe is best known to the public, are—"The Principles of Physiology applied to the Preservation of Health, and to the Improvements of Physical and Mental Education," of which twelve editions have been called for since its first appearance in 1834; "The Physiology of Digestion, considered with Relation to the Principles of Dietetics," originally published in 1836, and now in the seventh edition; and "A Treatise on the Physiological and Moral Management of Infancy, for the use of Parents," of which the first edition came out in 1840, and the sixth or People's edition, in June of the present year.

These works have been republished in the United States, and the first and third mentioned have gone through many editions. This last was edited by Dr. Bell in a manner that gave much satisfaction to the author. As it is the fashion, just now, with some of our home critics, and particularly with those who are quite innocent of any literary attempt themselves, to speak in terms of disparagement of American editions of English works, we shall quote the concluding paragraph of the introduction to the last edition of the "Treatise on Infancy:"—"Here I cannot resist the opportunity of expressing my grateful acknowledgments to Dr. John Bell, of Philadelphia, for the time and trouble which, amidst many pressing avocations, he so kindly and disinterestedly bestowed, not merely in superintending the re-publication of this work in the Uuited States, but in enriching it with many valuable notes, for the purpose of adapting it more completely to the domestic habits and wants of the Transatlantic public. To Dr. Blicker, of Copenhagen, I am also indebted for its appearence in a Danish translation."

The aim of Dr. Combe, in preparing these works, is well expressed in the following sentences, by the author himself. "In teaching dietetic rules and hygienic observances, therefore, the precepts delivered should be connected with and supported by constant references to the physiological laws from which they are deduced. Thus viewed, they come before the mind of the reader as the mandates of the Creator; and experience will soon prove, that, by his appointment, health and enjoyment flow from obedience, and sickness and suffering from neglect and infringement of them." That he was entirely successful in his estimate, both of the importance of the subjects, and of the manner in which they are best taught, is shown by, not merely the wide circulation, but the careful study of his different treatises.

Dr. Combe and his elder brother, Mr. George Combe, with whom in this country he is often confounded, were among the leading members of the Edingburgh Phrenological Society, instituted in 1820. He contributed two essays to the volume of Transactions, published by that body in 1824, and subsequently wrote many valuable papers in the *Phrenological Journal*, which was commenced in 1823, and now extends to twenty volumes. In 1831, Dr. Combe published "Observations on Mental Derangement; being an application of the Principles of Phrenology to the Elucidation of the Causes, Symptoms, Nature, and Treatment of Insanity." This work has long been out of print; the infirm health of the author having, as he tells us, prevented him from devoting that attention to the treatment of insanity, and, consequently, of "doing that justice to the subject, which its later progress and inherent importance imperatively demand." In the beginning of 1846, his strong conviction of the importance of phrenology to medical men, induced him to write, at the penalty of considerable fatigue, an "Address to the students of Anderson's University, Glasgow, at the opening of Dr. Weir's first course of lectures on Phrenology."* It was delivered to a crowded audience by his brother, (Mr. George Combe,) and subsequently appeared as a pamphlet. He also contributed several articles to the *British and Foeign Medical Review*, and was an occasional writer on medical and sanitary subjects in the columns of the *Scotsman*, a paper conducted with great ability and exerting no little political influence in North Britain.

From the biographical sketch of Dr. Combe, which appeared in the Scotsman of Aug. 21, we extract the following characteristic traits of the deceased, in the accuracy of which we have full reliance, For ourselves we cannot boast of the advantage of much personal intercourse with Dr. Combe; but, even from the slight acquaintance with him which his few days' visit to Philadelphia, in the early part of the last summer, allowed us to make, we are prepared to adopt the opinions of his friend in the paper just referred to.

"The decease of Dr. Combe, will have taken no one who knew him by surprise, for he was for many years in that condition, with the loss entirely of one lung, which makes life a greater miracle than death; but it will not on this account be the less deplored, either as causing a blank in the circle of private friendship, or as the signification of a public loss. Dr. Combe belonged to that rare class of physicians who present professional knowledge in connection with the powers of a philosophical intellect, and yet, in practical matters, appear constantly under the guidance of a rich natural sagacity. All of his works are marked by a peculiar earnestness, lucidity and simplicity, characteristic of their author; they present hygienic principles with a clearness for which we have no parallel in medical literature. To this must be ascribed much of the extraordinary success they have met with, and on this quality undoubtedly rests no small share of their universally acknowledged utility. Those, however, who look below the surface will not fail to trace a deep philosophical spirit, as pervading these works, something arising from a perfect apprehension of, and a perfect allegiance to the natural rule of God in our being. It has been a guidance, one would almost say an inspiration, of the author, without ever carrying him for a moment where ordinary readers could not follow him. Here we think is the true though latent strength of Dr. Combe's popular writings, and that which will probably give them a long, enduring preeminence in their particular department. We always feel, in reading them, that we are listening to one of those whom nature has appointed to ex-

*Printed in the London Lancet, and in the Bulletin of Medical Sciences, edited by Dr. Bell.

pound and declare her mysteries for the edification of her multitudinous family. In his own section of her priesthood, certainly few have stood in his grade, fewer still become his superiors.

"The personal character and private life of Dr. Combe formed a beautiful and harmonious commentary upon his writings. In the bosom of his family and the limited social circle to which his weakly health confined him, he was the same benignant and gentle being whom the world finds addressing it in these compositions. The same clear sagacious intelligence, the same entire right-mindedness, shone in his conversation. An answer to any query put to him, whether respecting professional or miscellaneous matters, was precisely like a passage of one of his books, earnest, direct and conclusive. Whatever measure he called upon others to do or to avoid, that he did, and that he avoided, in his own course of life; for doctrine, with him, was not to be treated as external to himself, but as the expression of a system of divine appointment, of which he was a part. To his rigid though unostentatious adherence to the natural laws which he explained, it was owing, that he sustained himself for many years in a certain measure of health and exemption from suffering, while labouring under the consumptive tendency which finally has cut short his career. On this point, there is the more reason to speak emphatically, when we reflect that the years thus redeemed from the grave were employed in that which will yet save many from premature death, as if it had been his aim to show the value of even the smallest remains of life and strength, and thus advance one of the principles dearest to humanity. It was not, however, in any of these respects that the character of Dr. Combe made its best impression, but in his perfect geniality and simplicity, and the untiring energy of his practical benevolence. Here resided the true charm of his nature, and that which made him the beloved of all who knew him. No irritability attended his infirm health; no jealousy did he feel regarding those whom superior strength enabled to outstrip him in the professional race. Kindly and cordial to all, he did not seem to feel as if he could have an enemy, and therefore we believe he never had one. It might almost be said that he was too gentle and unobtrusive—and so his friends, perhaps, would have thought him, had it not, on the other hand, appeared as the most befitting character of one who, they all knew, was not to be long spared to them, and on whom the hues of a brighter and more angelic being seemed already to be shed."

In a letter from a near relative of Dr. Combe, announcing the death of the latter, addressed to Dr. Bell, it is stated that Dr. C., after going to bed on the evening of the 2d of August, in his usual state of health, was seized with diarrhœa, under which, after ineffectual attempts to check it, he sank on the 9th of the same month. He had very little suffering, and his mind continued calm and cheerful to the last. Frequently, he expressed his thankfulness that he was permitted to depart so easily. The immediate cause of his death was ascertained, in *post mortem* examination, to be a "chronic disease of the bowels, terminating in ulceration." "The left lung was wasted away and completely useless; the right nearly, or altogether entire." For years past Dr. Combe knew that his left lung was gone; his chest on that side had sunk in. We shall probably yet receive a more detailed account of the structural changes from Dr. John Scott, who was his professional attendant and friend.

---

OBITUARY.

Died, on the 30th of August in the city of New York, James A. Washington, M. D., in the 46th year of his age. Dr. W. studied medicine in this city, and was sometime one of the resident physicians of the Pennsylvania Hospital. The following account of the post mortem examination of the body, we extract from the Annalist of the 15th ultimo. It will be read with a melancholy interest by his many friends in Philadelphia.

To the Editor of the Annalist.

I send you an account of the post-mortem appearances of our much-lamented medical brother, Dr. Washington, who has been so suddenly removed from among us, believing it will be interesting to your medical readers. At the request of Dr. Parker, I made the autopsy eighteen hours after death. Present, with Dr. Parker, Drs. Delafield and Borrowe.

*External Appearances.*—The body is not emaciated, but slightly jaundiced, and decomposition is commencing about the neck; on the abdomen are marks of leech-bites, and a blister.

*Head.*—Not examined.

*Chest.*—Heart, normal. Left lung:—upper lobe united by very old adhesions; the lung otherwise normal. Right lung:—in the summit of the upper lobe was found a small obsolete tubercle, about the size of a buck shot, while the posterior part of the lower lobe was congested.

*Abdomen.*—The subcutaneous cellular tissue loaded with fat about one inch in thickness. The muscular tissue was red and firm; on dividing the peritoneum, the omentum was found matted together, and lying over to the right iliac region, and adherent to the caput coli, where it formed part of the walls of a fœcal abscess, which was situated in the right iliac and lumbar regions; the remaining portion of the walls of this abscess was formed partly by the caput coli, folds of the ileum, and the peritoneal lining of the wall of the abdomen; it was lined by a recently formed grayish false membrane; the cavity was about the size of a hen's egg, containing a small quantity of light-brownish coloured feculent matter; at the lower part of the abscess, the appendix cæci vermiformis was found in a gangrenous condition, and on its careful removal, it was found obstructed by a small body about the size and shape of a bean, lodged about half an inch from its connection to the cæcum. Beyond this, the vermiform process was gangrenous; there was a small perforation at its extremity, from which the feculent matter had escaped. The mucous coat of the caput coli was red, thickened, and covered with small granules of lymph; the remaining portion of the colon was healthy. Several folds of the ileum and jejunum were adherent by recently formed lymph, but there was very little effusion into the abdominal cavity.

Stomach and spleen, normal.

Liver about its natural size, of a yellowish granular structure; the gall bladder was filled by dark, greenish, inspissated bile; gall ducts clear.

*Kidnies.*—The left enlarged, and in the early stage of granular disease; right, healthy.

Sincerely yours,

G. A. Sabine, M. D.

---

MEDICAL APPOINTMENTS.

The following appointments of Professors have recently been made:

*University of Pennsylvania.*—James B. Rogers, M. D., Professor of Chemistry, vice Professor Hare, resigned.

*Ohio Medical College.*—Professor L. M. Lawson, of the University Transylvania, Professor of Materia Medica, vice Prof. Harrison, transferred to the Chair of Theory and Practice of Medicine.

*Hampden Sidney College.*—Charles Bell Gibson, M.D., Professor of Surgery, vice Professor Warner, deceased.

*University of New York.*—Professor S. H. Dickson, of the Medical College of South Carolina, Professor of Theory and Practice, vice Professor Revere, deceased.

*College of Physicians and Surgeons of the city of New York.*—Professor Alonzo Clark, of Pittsfield, Lecturer on Physiology and Pathology.

*Medical College of South Carolina.*—Dr. Bellinger, Professor of Surgery, in the place of Professor Geddings, transferred to the Chair of Practice, vice Dr. Dickson, resigned.

*Philadelphia College of Medicine.*—Henry Gibbons, M. D., Professor of Institutes and Practice of Medicine, vice Prof. Thomas D. Mitchell, resigned. D. P. Gardiner, M. D., Prof. of Chemistry, vice Professor Allen, resigned. Louis H. Beatty, M. D., Professor of Obstetrics.

*Hampden Sidney College and University of Virginia.*—Professor Cabell, whose appointment to the Chair of Surgery in Hampden Sidney College was recently announced, declines the appointment, and retains his connexion with the University of Virginia.

### ANNUAL ANNOUNCEMENTS.

We have received the following Annual Announcements and Catalogues of Medical Colleges:

*Annual Announcement of the Medical Department of Illinois College Jacksonville, Ill. Session of* 1847–8.

This College has five professors. The lectures commence on the first Monday in November and continue four months. *Number of the last class,* 39; *of graduates,* 13.

*Fifth Annual Announcement for* 1847–48, *and Catalogue for* 1846–47, *of the Rush Medical College, Chicago, Ill.*

Rush Medical College has six Professors. The lectures commence on the first Monday of November, and continue *sixteen weeks.* Number of the class attending the last session, 70; of graduates, 16.

*Catalogue of the officers and students of the Medical Department of Hampden Sidney College, in Richmond, Virginia. Session of* 1846–47.

The Faculty consists of six Professors. The lectures commence on the first of November, and continue until the third week in March. Number of the class, 75; of graduates, 17.

*Annual Circular and Catalogue of the Medical Department of the University of Buffalo.*

Number of Professors, 7; of the class, 67; of graduates, 18.

The lectures commence on the last Wednesday in February, and continue sixteen weeks.

